# Mitochondrial metabolism in cancer stem cells (CSCs): molecular and diagnostic implications

**DOI:** 10.3389/fmolb.2026.1741800

**Published:** 2026-03-04

**Authors:** Charan PSVV, Sona Sunil, Nandana Thuyyath, Rekha Rani Kokkanti, Kousalya Lavudi

**Affiliations:** 1 Amrita School of Biotechnology, Amrita Vishwa Vidyapeetham, Kochi, India; 2 Amrita School of Nanosciences and Molecular Medicine, Amrita Vishwa Vidyapeetham, Kochi, India; 3 M. S. Ramaiah University of Applied Sciences, Bengaluru, India; 4 School of Biotechnology, KIIT Deemed to be University, Bhubaneswar, India; 5 Department of Radiation Oncology, The Ohio State University, Columbus, OH, United States

**Keywords:** CSCs, drug resistance, metabolic plasticity, mitochondrial bioenergetics, mitochondrial dynamics, OxPhos, therapeutic resistance, tumor relapse

## Abstract

Cancer stem cells (CSCs) are a self-renewing population often linked to tumor initiation, metastasis, relapse, and resistance to therapy. While bulk tumor cells are often dependent on glycolysis, CSCs demonstrate metabolic plasticity can switch between glycolysis and OXPHOS (oxidative phosphorylation) depending on context. Mitochondria buffer against stress and allow for a metabolic reprogramming towards apoptosis evasiveness, making mitochondrial function crucial to CSC survival. The acquisition of stem-like traits coincides with the rewiring of mitochondrial metabolism, as newly emerging CSCs intermittently upregulate respiration, ROS detoxification, and metabolic plasticity to satisfy cellular demands. Several regulators converge on this mitochondrial metabolism axis. For instance, the co-activator peroxisome proliferator-activated receptor gamma coactivator 1α (PGC-1α) and partner estrogen-related receptor α (ERRα) promote mitochondria biogenesis and OXPHOS while promoting tumor sphere formation and expression of stemness genes. Conversely, knockdown of PGC-1α reduces sphere formation and stemness. Similarly, a crucial process - mitophagy via AMP-activated protein kinase (AMPK) and related kinases regulate organelle turnover and quality control to promote CSC viability against stress. Mitochondrial dynamics (fission/fusion) also decides the fate of CSCs. The CSC metabolism is further influenced by the tumor microenvironment (TME). Hypoxia-inducible transcription factors, along with tumor stromal signals such as CAF-derived metabolites induce metabolic rewiring and strengthen antioxidant defenses in CSCs, thereby making it easier for CSCs to survive in unfavourable niches. The abundance of mitochondrial DNA and basal respiratory activity has been linked to CSC features such as increased ATP, stem cell markers and chemoresistance. Over the past few years, significant progress has been made in targeting mitochondrial metabolism of CSCs, yet is still a developing area with tremendous therapeutic scope. More research is required to identify mitochondrial vulnerabilities that are specific to therapy and then translate those findings into effective, precision-based cancer treatments. In this review, we try to provide a comprehensive overview of mitochondrial metabolism in regulating behaviour of CSCs, origin and characteristics of CSCs, the metabolic reprogramming for OXPHOS and glycolytic flexibility, molecular regulators of mitochondrial function, mitochondrial dynamics in stemness pathways and how the TME regulates these processes. We also review novel diagnostic techniques and therapies that target mitochondrial vulnerabilities to eliminate CSCs and provide better clinical outcomes.

## 1 Introduction

Cancer stem cells (CSCs) are rare and self-replicating subpopulations of cancer cells that initiate, maintain, promote tumor growth, and are responsible for treatment resistance. They possess the ability to self-renew independently and to generate a spectrum of cell lineages that make up a tumor (Chu et al., 2024). In principle, CSCs retain key features of normal stem cells such as self-renewal, differentiation into multiple lineages and establish intratumoral heterogeneity (Tang, 2012). This diversity imparted by CSC often aggravates proliferation and metastatic potential (Lee et al., 2025). CSCs are tumor initiating cells (TICs) in both local and metastatic tumors and often give rise to a spectrum of differentiated phenotypes. CSCs possess the ability to evade therapies through various mechanisms; however, this is partly due to their frequent over-expression of drug efflux transporters, which helps to remove drugs from the cytosol and DNA repair proteins, or simply enter a dormant/quiescent state that allows them to survive therapies directed toward the actively dividing cancer cell population (Kapoor-Narula and Lenka, 2022). As a result, once most of the tumor cells have been eliminated, the remaining CSCs can reinstate the tumor, which promotes the recurrence of the disease and drug resistance as seen with aggressive cancers (Bao et al., 2006; Doyle et al., 1998). Their quiescent state can evade standard treatments that can eliminate most dividing cancer cells and leave CSCs which eventually leads to tumor relapse. With developments in this regard, cancer stem cells are being viewed as a fundamental driver of cancer recurrence and now as potential therapeutic targets (Shen et al., 2020; Huang et al., 2020).

Metabolic heterogeneity and plasticity represent a defining hallmark of CSCs as they frequently exhibit diverse metabolic phenotypes. CSCs have been identified in almost every type of cancer, for instance, leukemia, breast, brain, colon and melanoma, and CSCs adopt unique metabolic programs appropriate for their cells of origin (Yadav and Desai, 2019). Unlike bulk tumor cells which significantly depend on aerobic glycolysis also referred to as “Warburg effect,” CSCs can adapt and utilize mitochondrial oxidative phosphorylation (OXPHOS), fatty acid oxidation and amino acid metabolism as metabolic substrates (Sancho et al., 2016; Viale et al., 2014; Lu et al., 2015). For example, during hypoxic conditions, they might selectively activate alternative pathways to sustain intracellular ATP and NADPH pools (Kuwabara et al., 2018; Xie et al., 2022). This metabolic flexibility promotes energy production, metabolism, and redox balance, as well as offers some protection from reactive oxygen species (ROS). CSCs potentially maintain proliferation and stemness by activating various metabolic pathways while bulk tumor cells cannot (Liu Y. et al., 2023).

This metabolic plasticity of CSCs is extensively dependent on mitochondria. The mitochondria produce ATP as an energy source in cells through the tricarboxylic acid (TCA) cycle and with the electron transport chain (ETC). Recent research highlights mitochondria as a signaling hotspot in cancer. Mitochondria also integrate signals required for cell survival, redox homeostasis, and apoptosis, and their ROS acts as a potent second messenger in these events (Du et al., 2025; Santidrian et al., 2013). Mitochondria generate metabolic resources for maintenance, coordinate the redox state of the cell through NADPH and ROS and they also modulate nuclear processes via bioenergetic and metabolic signaling. Although glycolysis is often prioritized in cancer, functional mitochondria remain important. For instance, Kidwell et al., demonstrated that transferred macrophage mitochondria often becomes destabilized and generates high levels of ROS, which eventually activates ERK signaling and drives proliferation, angiogenesis and immune evasion *in vitro* and *in vivo* (Kidwell et al., 2023). These functions are primarily salient with respect to CSCs, as the mitochondria not only fuel the stem cell like metabolism but also affect the stem cell associated gene regulatory networks.

Apart from metabolism, mitochondrial dynamics and turnover directly affect CSC behaviour and fate. Recent findings indicate that mitochondria are essential regulators of CSC survival and stemness (Egan et al., 2021). For instance, mitochondria often perform a vital role in the process of metabolic reprogramming that is important for CSC maintenance through NADH/NAD+ ratios adjustment and metabolic intermediates production. Mitochondrial metabolism feeds back on the nucleus as changes in the NADH/NAD+ ratio, acetyl-CoA, α-ketoglutarate (alpha-KG), and ROS can significantly modify epigenetic enzymes such as histone demethylases and histone deacetylases (HDAC), resulting in altered transcription of stemness genes (Etchegaray and Mostoslavsky, 2016). In general, CSCs display altered mitochondrial dynamics, fusion/fission, biogenesis and mitophagy, which sets them apart from differentiated tumor cells (Sessions and Kashatus, 2021).

Many studies have reported that CSCs often present altered mitochondrial morphology. For instance, an increase in mitochondrial biomass and hyper-fusion networks, a phenotype that is associated with increased OXPHOS. This increased activity is believed to play a crucial role in buffering capacity to metabolic stress, while also relating to invasive phenotypes (Liu Y. et al., 2023; Sessions and Kashatus, 2021; Farnie et al., 2015; Shin and Cheong, 2019). Conversely selective removal of mitochondria (mitophagy) is equally important and plays a vital role in maintaining low ROS in CSCs. Panigraphi et al. observed that in CSCs, mitophagy enables plasticity since it rewires metabolism to favor the tumor niche (Panigraphi et al., 2020). In terms of therapy, mitochondria remains a target of interest in CSCs. To selectively eliminate CSCs multiple alternative strategies have been evaluated such as oxidative phosphorylation inhibitors, and other agents that disrupt metabolic pathways of the mitochondria (Tan et al., 2025). Zheng et al. surveys multiple preclinical/clinical targeted and nanoparticle interventions against CSCs (Zheng et al., 2023) in a recent review. Engineered nanocarriers can be modified with ligands that will direct them toward CSCs, such as antibodies that bind to CD44 or CD133. For instance, nanoparticle formulations of OXPHOS inhibitors such metformin, and oligomycin that preferentially accumulate in CSC-rich tumors located in the hypoxic niche of the tumor and significantly increase drug uptake by CSCs. The development of liposomal and polymeric nanoparticles containing glycolysis inhibitors and fatty-acid oxidation inhibitors which preferentially accumulate in CSCs when they are functionalized with relevant targeting peptides (Li et al., 2024; Xiong et al., 2016; Chen et al., 2015). In this area of research, many recent reviews indicate that mitochondrial targeted nanomedicines represent a promising research area. Specifically, several recent reviews have highlighted dendrimer and polymer-based carriers that can be used on the higher membrane potentials of CSC mitochondria to deliver drugs directly into the mitochondrial organelle. For example, carbon-based nanocarriers loaded with metformin could be used to selectively enhance the effects of metformin on hepatocellular CSCs and increase their rate of death (Mazloomi et al., 2025). In conclusion, emerging evidence shows that mitochondria are master regulators of CSC fate. CSCs survival and maintenance depend heavily on mitochondrial metabolism and signaling pathways. The altered mitochondrial metabolism represents a potential vulnerability and can pave a way for the future precision cancer therapies.

## 2 Overview of cancer stem cells

CSCs were first identified in malignancies across various tumors including breast, brain, colon and pancreatic cancers (Batlle and Clevers, 2017; O’Brien et al., 2007). These reside at the top of the tumor hierarchy and are responsible for initiation, progression, metastasis, and recurrence after therapy. The concept of CSCs has dramatically impacted our comprehension of tumor biology. The origin of CSCs is still debated whether they originate from normal stem or progenitor cells that have become transformed, or from differentiated cells that have become reprogrammed (Scheel and Weinberg, 2011). Considering CSC markers, only certain cells may be marked with CSC markers. The presence of CSCs is significant for how tumors behave and respond to therapy. CSCs are thought to underlie tumor heterogeneity and treatment resistance due to their relatively continuous production of new cells (Kuşoğlu and Biray Avcı, 2019). For instance, tumor cells from patients with chemotherapy or radiation therapy, residual tumors, have been shown to have a larger proportion of CSC testable markers (Kurrey et al., 2009). Resistance to therapy is likely to be due to several characteristic features of CSCs. Mainly, CSCs can be relatively quiescent compared to the bulk tumor, which could make them immune to therapies that attempt to inhibit the proliferative potential of the tumor (Li, 2013).

**FIGURE 1:**
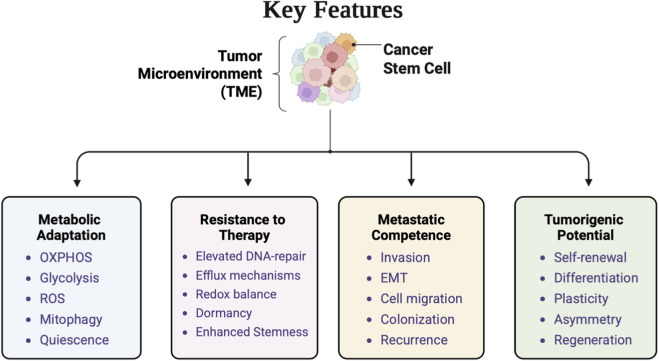
Figure represents the key features of CSCs.

CSCs are resistant to exposure to chemotherapy and radiation as they mostly remain inactive and have high DNA repair efficiency, and pump out drugs via unique transporter proteins (Hanh Phi et al., 2017). The cells express various surface markers such as CD44^+^/CD24^−^, CD133^+^, and ALDH1^+^,which are specific to different subtypes and microenvironment. Functional validation of CSC properties is achieved through assays including tumor sphere formation, serial transplantation, and resistance profiling. These approaches confirm their capacity for unlimited self-renewal and tumor initiation, distinguishing CSCs from bulk tumor cells. At the molecular level, CSC maintenance and survival are governed by signaling networks that overlap with embryonic development pathways, including Wnt/β-catenin, Notch, Hedgehog, and PI3K/Akt/mTOR (Takebe et al., 2015; Koury et al., 2017; Borlongan and Wang, 2023; Lee et al., 2025). Dysregulation of these cascades leads to constitutive activation of transcription factors such as SOX2, NANOG, and OCT4, which sustain stemness and inhibit differentiation. Moreover, epigenetic modulators such as histamine methyltransferase and DNA demethylases contribute to maintaining CSC plasticity, enabling dynamic transitions between stem-like and differentiated states (Liu et al., 2013; Galassi et al., 2025; Kumar et al., 2022; French and Pauklin, 2021; Zhang and Zhang, 2024) (Figure 1).

It is increasingly appreciated that the identity of CSC is also closely associated with metabolic reprogramming. CSCs possess high metabolic flexibility and a capacity to fluctuate between glycolysis and OXPHOS in accordance with external stimulation and nutrient supply (Vlashi and Pajonk, 2015). This metabolic flexibility not only provides for their biosynthetic and redox needs but also serves as the basis of their stress survival, protection against oxidative damage, and therapy resistance. As a whole, they are the functional center of tumor heterogeneity and resistance. Revealing their regulatory signals, epigenetics, and metabolic dependencies is critical for designing targeted therapies. Emerging treatment strategies now focus on specifically targeting CSCs, as perturbation of their population is necessary for long-term remission and to prevent relapse (Dzobo et al., 2020). The hallmark features of CSCs and their functional significance is depicted in Table 1.

**TABLE 1:** Hallmark features of CSCs and their functional significance.

Feature	Key insights	Functional significance
Hierarchy and self-renewal	CSCs occupy the apex of the tumor hierarchy and are uniquely self-renewed to regenerate the entire tumor population	Maintains tumor growth and cellular heterogeneity; central to long-term cancer propagation
Differentiation potential	CSCs generate more differentiated progeny that typically lack tumorigenic capacity	Creates phenotypic diversity within tumors; links stemness to tumor heterogeneity
Tumor initiation	Only CSCs can serially initiate tumors in immunodeficient mice, fulfilling the defining criterion of tumorigenicity	Establishes CSCs as the principal drivers of tumor initiation and recurrence
Marker profiles	Express variable stem-associated markers (e.g., CD133, CD44, ALDH); marker sets differ by cancer type	Enables CSC identification and isolation; highlights need for tumor-specific marker panels
Therapy resistance	Exhibit quiescence, robust DNA repair, and antioxidant mechanisms that confer treatment tolerance	Promotes therapy evasion and disease relapse; necessitates CSC-targeted therapeutic approaches
Metastatic potential	CSC-like cells mediate invasion and colonization of distant sites via enhanced survival and adaptability	Facilitate metastatic spread and likely represent metastasis-initiating cells

In conclusion, CSCs are a unique tumor cell compartment that is paramount for cancer progression and for maintaining tumor heterogeneity. In seeking to establish more effective therapies for eliminating tumor growth, the field of research focusing on the biology of CSCs remains to identify, target and understand their vulnerabilities (Chen et al., 2003).

## 3 CSC-TME interactions

**FIGURE 2:**
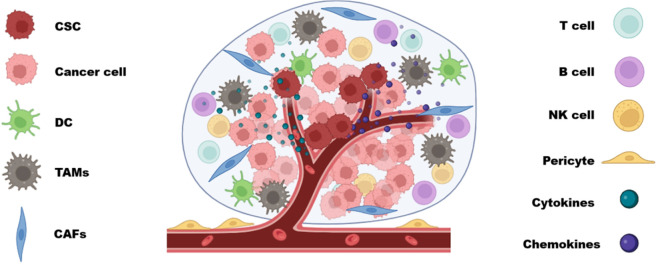
Diagrammatic representation of TME and the interacting components including Immune cells (T cells, B cells, NK cells and Dcs), Stromal components (CAFs, TAMs) along with CSC and cancer cells.

The TME represents the immediate surroundings of CSCs, which comprise cellular components such as niche cells, and non-cellular elements such as growth factors and ECM. The TME undergoes constant evolution based on the behavior of the tumor, and its exposure to treatment strategies, enabling it to constantly adapt and alter itself to resist the changes by the immune response and therapeutic intervention. The main cellular components recognized from the TME involve the pericytes, CAFs, macrophages, bone marrow derived mesenchymal stem cells (BM-MSCs), adipocytes and immune cells that undergo transitioning from its normal phenotype into tumor promoting phenotype, based on the soluble factors, cytokines, and growth factors received from the TME (Figure 2) (Yin et al., 2021). The TME plays a crucial role in sustaining and nourishing the CSCs population analogous to the maternal environment protecting a developing fetus. It supplies the CSCs with an ideal environment that promotes the stemness and plasticity properties, thereby allowing the tumor to metastasize and evade the immune system (Castelli et al., 2021). Hence, in other words, the survivability of the CSCs solely depends upon their interaction with the soluble factors and cellular components present in the TME. This interplay between the TME and CSCs is one of the crucial aspects that promote tumor metastasis and insensitivity to current therapies. Current research highlights the importance of targeting the CSCs along with their TME (Xiao and Yu, 2021). It is considered to be a promising approach, as the destruction of the TME and CSC specific niche will lead to the depletion of the necessary cues and signals required by the CSCs for their survival. However, the existing tumor cell heterogeneity and tumor variability between patients may play a significant role in its therapeutic resistance (Bayik and Lathia, 2021).

The occurrence of tumor infiltrating cells have been documented for multiple cancer types and include the intrusion of myeloid deprived suppressor cells (MDSCs), macrophages, granulocytes, dendritic cells (DCs), lymphocytes, neutrophils, regulatory T cells (T regs), cytotoxic and T helper cells (Th). All these cells are found to interact with CSCs and function based on the cues received from TME (Figure 2) (Bayik and Lathia, 2021). Tumor associated macrophages (TAMs) are one of the significant cell populations observed within the tumor microenvironment (Figure 2). They originate either from the differentiation of immature monocytes or by the phenotype conversion of tissue resident macrophages. The polarization of the macrophages resident in the TME into inflammatory or immune suppressive roles depends on the necessities of the growing tumor (Bayik and Lathia, 2021; Cheng et al., 2022). The expression of C-C motif chemokine 2 (CCL2) and macrophage colony-stimulating factor 1 (M-CSF) within the TME, are known to play pivotal roles in regulating the infiltration and the differentiation of monocytes into TAMs (Qian et al., 2011). This transition of infiltrating monocytes and tissue resident macrophages into TAMs is essential for activating neoangiogenesis, uncontrolled tumor cell proliferation, EMT, and to induce or sustain the immunosuppressive state of the TME (Basak et al., 2023). A dual crosstalk between CSCs and TAMs occur, wherein TAMs facilitate the maintenance of the CSCs and promote its stemness through various factors such as WNT ligands, pleiotrophin, TGF-β and Interleukin-6 (IL-6) (Basak et al., 2023; Shi et al., 2017; Wan et al., 2014; Fan et al., 2014). The CSCs in return help the TAMs to evade the immune attack and thereby preserve the TAMs and its subpopulations to prolong the immunosuppressive nature of TME (Basak et al., 2023).

DCs are a subset of immune cells that function as Antigen presenting cells to Th cells and to engage in immune response, thus leads to altered function in aiding immune inhibition due to the signals secreted by the TME (Figure 2). The immunosuppressive stimuli present in the TME allows for the conversion of infiltrated DCs into tolerogenic DC subtypes, and may alternatively inhibit the recruitment and the maturation of DCs. In addition to the suppression of DC regulated immune response, the tolerogenic DCs contribute in enhancing tumor progress and metastasis and influences the reprogramming of Th cells. Hence, the CSCs and TME indirectly benefit from DC reprogramming, by triggering the remaining immune population to adapt tumor promoting traits (Engblom et al., 2016; Kenkel et al., 2017; Barilla et al., 2019). Recent studies suggest the role of tumorigenic DCs in facilitating the retention of CSCs in ovarian cancer, breast cancer, colorectal cancer, melanoma etc (Del Prete et al., 2023).

The emerging research suggests that the stromal cytokines can alter the metabolism of CSCs directly. For example, IL-6 produced by MSCs has been shown to increase the transfer of mitochondria from the stroma to the leukemia cells (Hou et al., 2025). In a comprehensive study, genetic ablation of IL-6 produced by mesenchymal stromal cells (MSCs) eliminated mitochondria transfer from MSCs to acute myeloid leukemia (AML) cells and significantly decreased their OXPHOS and restored chemosensitivity (Ahmed et al., 2025). In this case, IL-6 from MSC provides the cellular machinery for the CSC mitochondria to be functional, increasing OXPHOS and drug resistance. The cytokines activate the CSC signal transduction pathways (STAT3/NF-κB), resulting in increased production of mitochondria, antioxidants, and glycolytic capacity (Singh et al., 2023). For example, IL-6/STAT3 signaling induces the expression of mitochondrial regulators and glycolytic enzymes that leads to increased stemness (Ando et al., 2010; Carbognin et al., 2016). According to a recent study Yousif et al., dendritic cells have been identified as a major source of the soluble IL-6 receptor, which acts as a buffer in the blood for regulating IL-6 levels and regulating the systemic inflammatory response, which provides a means of modulating hyperinflammation and improving IL-6-targeted therapies (Yousif et al., 2021). Collectively, the above observations suggest that cytokines produced in the CSC niche, particularly IL-6 are the reason for the transfer of mitochondrial and metabolic reprogramming of CSCs, resulting in the survival and drug resistance of CSCs which are driven by OXPHOS (Kesh et al., 2020).

MDSCs are a group of cells derived from the myeloid lineage via the immature differentiation of bone marrow derived cells, and are generally of two subpopulations; monocytic MDSCs (M-MDSCs) and polymorphonuclear MDSCs (P-MDSCs) (Veglia et al., 2021). Another group of tumor-associated neutrophils (TANs) have been observed to have a similar profile as that of P-MDSCs (Ouzounova et al., 2017b). The MDSCs are increasingly being detected as one of the characteristic cells in the CSCs niche, inducing an immunosuppressive environment, inhibiting the antitumor response by directly involving with the innate and adaptive immune response (inhibiting T cells, B cells and NK cells) and maintains a highly oxidative stress in the TME (Figure 2). They are also known to positively impact the production of Tregs and TAMs (Zhao et al., 2023). MDSCs are also known to enhance cancer progression and metastasis and may negatively affect the current immunotherapies (Liu et al., 2018). It has been demonstrated that in the presence of IL-6 and GM-CSF, which can be influenced by STAT3 pathway, has known to increase the immunosuppressive nature of MDSCs, promote its infiltration, inhibit CD8^+^ T cells and can negatively control the interferon regulatory factor (IFN-8) (Mojsilovic et al., 2021; Weber et al., 2020; Lechner et al., 2010; Waight et al., 2013).

Tregs are a subpopulation of T cells, involved in regulating extensive immune responses produced by the body. They are triggered in the presence of IL-2 and FOXP3 expression and act as prime regulators by inhibiting the Th, cytotoxic T cells and inhibit the differentiation of B cells (Sakaguchi et al., 2007; Brunkow et al., 2001; Grover et al., 2021). They do so through direct cell to cell or cell independent interactions, by CTLA4/PD1-PDL1 interactions (Leach et al., 1996; Ishida et al., 1992) and by upregulating the levels of immunosuppressive molecules such as IL-10, TGF-β (Grover et al., 2021). In tumors, CSCs attract the Tregs towards the TME, by releasing the chemokines such as CCL1, CCL2 and CCL5 (Chang et al., 2016; You et al., 2017; Xu et al., 2017). The cells carrying out the immune surveilance, CD4^+^ T cells, CD8^+^ T cells, B cells and NK cells are also being influenced by the cues in TME, such that the CSCs are able to escape their attack. The CSCs manipulate the immune cells by downregulating the expression of major histocompatibility complex class I (MHC-1) and NK cell receptor D ligand (NKG2D). The cytotoxic T cells require the MHC-1 to recognize the tumor antigens and generate an immune response, and in the absence of MHC-1, the T cell mediated immune response cannot be employed against CSCs. The NK cells are potent enough to identify MHC-1 deficient cancer cells, but are incapable of generating a response due to the lack of NKGD2 ligand (Sari and Rock, 2023; Jones et al., 2022). In spite of the ongoing research focussed on the role of TME in maintaining CSCs population, promoting cancer progression, and evasion of immune attack, there is still a lot more to understand regarding the complexities and intricacies of the TME.

## 4 Mitochondrial metabolic reprogramming in CSCs

**FIGURE 3:**
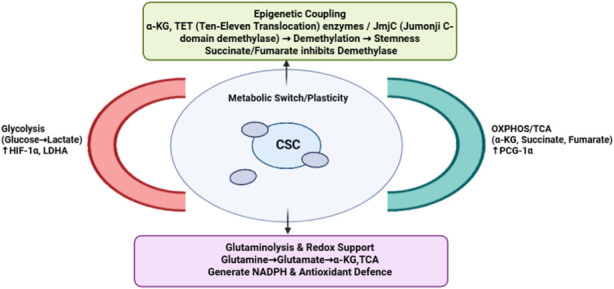
The figure depicts mitochondrial metabolic plasticity in CSCs and a dynamic metabolic switch between glycolysis and OXPHOS to adapt to changing energy and oxygen conditions to regulate stemness.

Mitochondrial metabolic reprogramming has emerged as a central hallmark of CSCs, enabling them to survive hostile tumor microenvironments, sustain self-renewal, and drive therapeutic resistance. While the Warburg effect historically emphasized the reliance of cancer cells on aerobic glycolysis, a growing body of evidence reveals that CSCs are not bound to a single metabolic phenotype but instead display remarkable plasticity, dynamically shifting between glycolysis and OXPHOS depending on context (Warburg, 1956; Vlashi and Pajonk, 2015). This metabolic adaptability distinguishes CSCs from bulk tumor cells, as it equips them with a flexible bioenergetic toolkit that can be engaged to maintain survival, proliferative potential, and tumor-initiating capacity in fluctuating environments (Viale et al., 2014). Unlike non-stem tumor cells that typically exhibit a glycolytic bias, CSCs frequently display an OXPHOS-dependent phenotype, with high mitochondrial respiration rates, elevated spare respiratory capacity, and greater mitochondrial mass (Janiszewska et al., 2012; Lamb et al., 2015). Glioblastoma CSCs, for instance, show higher oxygen consumption rates and rely on mitochondrial respiration for their clonogenicity, whereas differentiated glioblastoma cells favor glycolysis (Vlashi et al., 2011). Similarly, breast cancer CSCs depend on mitochondrial biogenesis orchestrated by PGC-1α, which drives OXPHOS activity and stemness. These findings challenge the traditional Warburg-centric model by demonstrating that stem-like tumor cells often favor oxidative metabolism. Yet, this reliance on OXPHOS is not absolute. Pancreatic CSCs, for example, adopt a hybrid metabolic state in which both glycolysis and mitochondrial respiration are simultaneously active, enabling them to flexibly switch depending on nutrient availability or therapeutic stress (Sancho et al., 2016). This duality underscores the concept of metabolic plasticity, whereby CSCs maintain the ability to transition between metabolic programs as survival requires (Peiris-Pagès et al., 2016; Martínez-Reyes and Chandel, 2021) (Figure 3).

The biosynthetic dimension of mitochondrial metabolism is equally critical to CSCs. Mitochondria act not only as ATP-generating organelles but also as hubs for anabolic precursor production. Glutamine metabolism plays a particularly central role. Through glutaminolysis, glutamine replenishes the TCA cycle and supports NADPH production, which maintains redox balance (Ward and Thompson, 2012; Altman et al., 2016; Ahn and Metallo, 2015). CSCs often exhibit glutamine addiction; for example, breast and pancreatic CSCs show heightened glutamine dependency, and inhibition of glutaminase suppresses their sphere-forming capacity and tumorigenicity (Leone et al., 2019; Ahn and Metallo, 2015). Beyond serving as fuel, mitochondrial metabolites such as alpha-KG, succinate, and fumarate exert profound effects on CSC biology by influencing epigenetic regulation. α-ketoglutarate acts as a cofactor for TET family DNA demethylases and Jumonji-C (JmjC) histone demethylases, linking mitochondrial metabolism to chromatin remodeling and gene expression (Carey et al., 2015). Conversely, accumulation of succinate and fumarate, often observed in tumors with mutation in SDH or FH, inhibits these dioxygenases, leading to epigenetic dysregulation that favors stem-like programs (Sciacovelli et al., 2016; Letouzé et al., 2013). Thus, mitochondrial metabolic rewiring directly impacts CSC fate by coupling intermediary metabolism with transcriptional and epigenetic landscapes.

**FIGURE 4:**
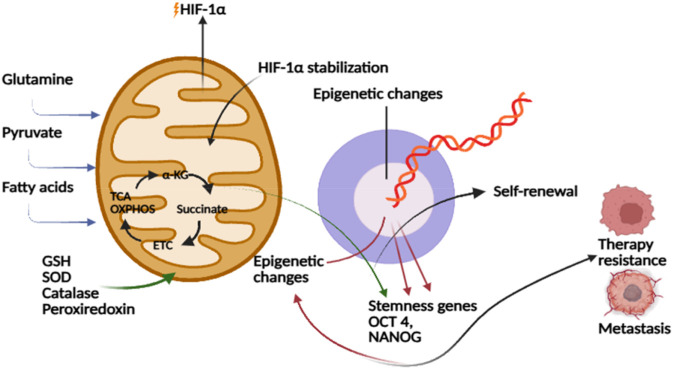
Mitochondrial metabolism regulates HIF-1α stabilization and epigenetic changes, promoting stemness gene expression (OCT4, NANOG), self renewal, therapy resistance, and metastasis.

Mitochondrial metabolic reprogramming in CSCs plays a vital role in redox homeostasis by enhancing antioxidant capacity. CSCs also tightly regulate ROS levels through robust antioxidant systems, including glutathione, superoxide dismutase, catalase, and peroxiredoxins (Diehn et al., 2009). ROS generated by mitochondrial electron transport chain activity provides another axis of reprogramming. ROS functions as a double-edged sword: excessive ROS damages macromolecules and induces differentiation or apoptosis, whereas moderate ROS levels act as signaling molecules that promote stemness (Diehn et al., 2009; Ishikawa et al., 2008). Mitochondrial ROS stabilize hypoxia-inducible factor-1α (HIF-1α), thereby enhancing glycolytic flux and promoting adaptation to hypoxic niches (Chandel et al., 2000; Semenza, 2012). ROS produced by radiolysis of water contribute to secondary, indirect oxidative damage, whereas direct ionization and oxidation of DNA molecules are the main ways that radiation exposure damages DNA. As stated by Diehn et al. (2009), CSC radioresistance is associated with reduced accumulation of radiation-induced DNA damage, even while they maintain moderate baseline ROS levels through increased antioxidant capacity. Thus, the survival advantage seen in CSCs after exposure to radiation is partly explained by ROS-mediated oxidative damage, though not entirely. CSCs have decreased ROS levels than non-stem tumor cells in breast cancer models, promoting redox balance but works in coordination with the predominant method of DNA-damage prevention during radiation response (Diehn et al., 2009; Wang S. et al., 2024). Such fine-tuned ROS regulation highlights how mitochondrial reprogramming integrates redox balance with maintenance of stem-like traits (Figure 4).

Mitochondrial structure and dynamics further contribute to CSC adaptation. Mitochondrial fission and fusion regulate not only morphology and optic atrophy 1 (OPA1), support elongated mitochondrial networks with high OXPHOS activity, while fission, mediated by dynamin-related protein 1 (DRP1), produces fragmented mitochondria associated with glycolysis and proliferation (Chen and Chan, 2009; Wai and Langer, 2016; Liu Y. et al., 2023). CSCs from breast and ovarian cancers frequently exhibit elongated mitochondria, consistent with OXPHOS dependency (Peiris-Pagès et al., 2016). On the other hand, enhanced DRP1-mediated fission supports glycolytic reprogramming and stemness in glioblastoma and leukemia (Xie et al., 2015; Katajisto et al., 2015). Mitophagy, the selective clearance of dysfunctional mitochondria, also contributes to CSC longevity by maintaining a healthy mitochondrial pool. These dynamic processes highlight that mitochondrial metabolism in CSCs is actively sculpted by organelle remodelling, ensuring functional adaptation to stress.

The metabolic characteristics of CSCs are widely influenced by the composition of their surrounding tissues (Yadav et al., 2020). For instance, when occurring in an adipocyte rich tissue such as the breast, CSCs use nearby adipocytes fat cells to provide the fatty acids required for metabolic demands (Chu et al., 2024; Masoudi et al., 2024). In contrast, when CSCs are present within a hypoxic niche like bone marrow due to limited oxygen availability, they are reliant upon neighbouring mesenchymal stem cells to provide support through the activation of OXPHOS and antioxidant pathways. In addition, CSCs residing within highly oxygenated or lipid-rich organs such as lung, liver, or brain, they can utilise alternative substrates, including neuron-derived lipids and ketone bodies in the brain, to sustain mitochondrial metabolism (Khaledian et al., 2023; Yao and He, 2021). Therefore, the environmental factors in different organs (oxygenation levels, nutrient availability, stroma composition) create unique metabolic demands for CSCs. Therefore, regardless of niche, CSCs opportunistically redirect mitochondrial metabolism to utilise the most abundant type of substrate present in that particular microenvironment (Wang et al., 2018; Snyder et al., 2018).

The therapy resistance of CSCs is closely linked to mitochondrial metabolic flexibility. Leukemia stem cells are highly OXPHOS-dependent and particularly vulnerable to mitochondrial complex I inhibitors such as IACS-010759 and venetoclax (Lagadinou et al., 2013; Pei et al., 2018). However, in solid tumors, metabolic adaptability allows CSCs to evade such targeting by switching between glycolytic and oxidative states. Radiation therapy, which elevates ROS levels, tends to enrich CSCs with strong antioxidant defenses, while chemotherapy often selects for OXPHOS- driven CSCs that can survive cytotoxic damage (Viale et al., 2014; Vlashi and Pajonk, 2015). This resilience reflects the fact that targeting a single metabolic pathway is insufficient to eradicate CSCs. Indeed, clinical observations confirm that while glycolysis inhibitors may reduce tumor bulk, they often fail to eliminate CSC populations, which compensate by shifting to mitochondrial respiration (Sancho et al., 2016). Thus, therapeutic resistance is intrinsically tied to the metabolic reprogramming capacity of CSCs. Notably, according to different tumor types and in the same tumor, mitochondrial reprogramming at the CSC level is highly heterogeneous. Recent work has highlighted breast cancer CSCs for having both an increased abundance of mitochondria and reliance on OXPHOS (Sancho et al., 2016), this intratumoral and intertumoral metabolic heterogeneity renders CSCs difficult to target, as no single metabolic inhibitor can uniformly suppress CSC populations across different cancers or even within the same tumor. However, it also indicates that context-specific vulnerabilities might be exposed through personalized metabolic profiling of CSCs (Table 2).

**TABLE 2:** Comparative overview of key metabolic features distinguishing CSCs from NSCs, highlighting shared stemness related traits versus CSC specific metabolic adaptations.

Metabolic feature	Cancer stem cells	Normal stem cells (NSC)	References
Glycolysis/PPP	CSCs frequently exhibit elevated glycolysis (Warburg-like) driving PPP flux for NADPH and nucleotide synthesis; it may support pluripotency and treatment resistance	NSCs mostly prefer glycolysis in hypoxic stem niches, providing quick ATP and biosynthetic precursors for self-renewal	Yadav et al. (2020), Stouras et al. (2023)
Mitochondrial dynamics and plasticity	Highly plastic metabolism: CSCs can switch between glycolysis and OXPHOS depending on conditions. Often exhibit elevated mitochondrial biogenesis (PGC-1α driven) and active mitophagy to maintain stemness and limit ROS.	Metabolic state linked to differentiation stage: quiescent NSCs have few, perinuclear mitochondria and limited flexibility. Upon differentiation, mitochondrial biogenesis and OXPHOS increase	Sancho et al. (2016), Fontana et al. (2024), Fu et al. (2019), Wanet et al. (2015)
Lipid metabolism and storage	CSCs posses enhanced fatty acid β-oxidation and desaturation to generate NADPH/ATP; accumulate lipid droplets and upregulate lipogenic enzymes (for instance, fatty acid synthase - FASN, and stearoyl-CoA desaturase - SCD1) to sustain stemness	NSCs often have an active *de novo* lipogenesis mediated by FASN/ACC - acetyl-CoA carboxylase activity and cholesterol synthesis support membrane biogenesis and pluripotency. Lipogenesis is necessary for stem cell maintenance	Mukherjee et al. (2017), Knobloch et al. (2013), Liu Z. et al. (2023)
Autophagy and nutrient scavenging	Upregulated autophagy and mitophagy to recycle intracellular nutrients, maintain mitochondrial quality, and survive metabolic stress and nutrient deprivation	Basal autophagy supports cellular homeostasis; extensive nutrient scavenging is uncommon under physiological conditions	Liu et al. (2019), Smith and Macleod (2019), Praharaj et al., (2022)
Dependence on niche and cell-intrinsic programs	Predominantly oncogene-driven and cell-intrinsic, allowing CSCs to survive outside normal stem cell niches and during metastasis	Largely dependent on niche-derived cues such as hypoxia, cytokines, growth factors to maintain metabolic state and stem cell identity	Snyder et al. (2018), Masoudi et al. (2024), Li et al. (2023), Zhang and Sadek (2014)
Redox homeostasis	Maintain exceptionally low ROS through enhanced antioxidant systems for example through glutathione synthesis, CD44–xCT axis, FOXO signaling, conferring resistance to oxidative stress and therapy	Low ROS maintained primarily through hypoxic niches and glycolytic metabolism; antioxidant mechanisms support quiescence but are less amplified than in CSCs	Ryoo et al. (2016), Nagano et al. (2013), Lendeckel and Wolke (2022), Sinenko et al. (2021), Urao and Ushio-Fukai (2013)

Taken together, mitochondrial metabolic reprogramming is a critical regulator of CSC biology. Through context-specific modulation of oxidative and glycolytic pathways, the preservation of biosynthetic and redox capacities, the remodeling of mitochondrial dynamics, and the assimilation of inputs from microenvironmental cues, CSCs establish a metabolic state that is both robust and adaptable. This property also enables them to survive therapeutic pressure, escape from clearance and cause relapse. Meanwhile, it is the key to finding the vulnerable points for therapeutic intervention. This type of combinatorial approaches targeting OXPHOS, glycolysis and antioxidant systems could be promising to overcome CSC survival and relapse (Martínez-Reyes and Chandel, 2021). Further studies that untangle the exact drivers of mitochondrial metabolism in CSCs will be essential not only to refine our mechanistic knowledge, but also for devising clinically relevant strategies that can robustly undermine the metabolic substrate supporting cancer stemness.

## 5 Molecular regulators of mitochondrial function in CSCs

The metabolic plasticity of CSCs relies significantly on the capacity of mitochondria to adapt to various forms of stress from the microenvironment and also the pressures from therapy. A recent study on mitochondrial signaling in CSCs highlighted that PGC-1α is one of the master regulators of mitochondrial biogenesis by co-activating nuclear respiratory factors (NRF-1 and NRF-2), and mitochondrial transcription factor A (TFAM). Alongside these lines, the process is also modulated largely by ERRα and AMPK (Praharaj et al., 2022). Another study revealed that the mTOR pathway antagonises biogenesis which illustrates how CSCs can utilize different levels of upstream pathways to reshape their mitochondrial network (Katsuno et al., 2019). PGC-1α plays a dual role, both as a biogenesis factor and also as a crucial mediator of OXPHOS. According to a recent study, the decrease of PGC-1α expression in CSCs resulted in a decrease in mitochondrial biogenesis and prevented proper OXPHOS, leading to a decrease in both ATP levels and metastasis (Fontana et al., 2024; LeBleu et al., 2014). This evidence shows that CSCs require PGC-1α so that they are able to increase their mitochondria mass and thereby reach metabolic demands for their survival, motility, and invasion during metastasis. Therefore, both mitochondrial biogenesis and bioenergetics are coupled to support the metabolic nature of CSCs to maintain their metastatic state (Liu et al., 2022).

Another such study demonstrated that ERRα forms a complex with PGC-1α to activate mitochondrial genes and simultaneously AMPK activates PGC-1α while mTOR suppresses PGC-1α to maintain the equilibrium between anabolic and catabolic signals (Luo et al., 2016). It has also been found that sirtuins like SIRT1 and SIRT3 deacetylate PGC-1α, increasing bioenergetics and causing CSCs to rely on OXPHOS or glycolysis (Koh et al., 2023).

A recent review by Garimella et al. highlighted that KRAS and c-MYC upregulate PGC-1α and mitochondrial biogenesis, resulting in OXPHOS and ATP production in CSCs. Cancer cells with high sphere-forming capacity often have lower expression of TFAM and reduced mtDNA, which shows that restricting biogenesis may result in stemness (Garimella et al., 2023). The knockdown of TFAM enhances sphere formation, whereas treatment with the mtDNA depleting agents such as ethidium bromide (EtBr) induces proliferation and expression of CSC markers. In this regard, oncogenic drivers such as KRAS and c-MYC can modulate mitochondrial activity into a hyperactive state instead of quiescence. The dichotomy of mitochondrial biogenesis in stemness is further indicated by the fact that some CSCs have low mitochondrial content to remain quiescent (Jin et al., 2022). Mitochondrial morphology is tightly regulated by fission and fusion processes, which can directly impact metabolism and CSC function (Liu Z. et al., 2023). A study about mitochondrial dynamics indicates that fission occurs when DRP1 is recruited to the fission site, facilitated by GTP hydrolysis. Mitochondrial fission is induced with phosphorylation at Ser616, and is inhibited with phosphorylation at Ser637 (Ingerman et al., 2005). In contrast, outer-membrane fusion is mediated by mitofusins 1 and 2 (MFN1/2), while OPA1 mediates inner-membrane fusion, resulting in either fragmented or fused networks of mitochondria (Chen et al., 2003). Structural fragmentation caused by excessive fission will induce glycolytic metabolism with excess ROS, whereas a fused mitochondrial network will support OXPHOS and ATP (Chen and Chan, 2017). This transition towards fissions is linked to increased stemness and resistance towards therapy. In contrast, elevated fusion often reduces CSCs viability.

**FIGURE 5:**
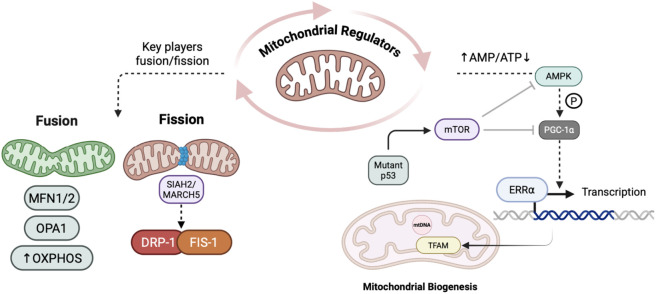
Figure represents various regulators of mitochondrial fusion/fission and role of AMPK/PGC-1α pathway and its regulatory networks supporting mitochondrial biogenesis/function in response to intracellular nutrient and energy deprivation.

Certain upstream kinases and ubiquitin-like ligases largely regulate mitochondrial dynamics by phosphorylating various proteins. Kinases such as ERK1/2, PKA phosphorylate DRP1 either in the activating or inhibiting site to fine regulate the fission rate (Senft and Ronai, 2016). Importantly, DRP1-FIS1 interaction plays a significant role in hypoxic stress, and this interaction is facilitated by ubiquitin ligases such as SIAH2 (Seven-in-absentia homolog 2) and MARCH5 (Membrane-associated ring Finger 5 E3 ubiquitin ligase.). Mitochondrial dynamics and stress response is largely influenced by MARCH5, as evidenced by a knockout study, which resulted in hyperfused or fragmented mitochondria and affecting activation of NF-*κ*B pathway (Nagashima et al., 2014). These alterations facilitate CSCs to rapidly modify their mitochondrial networks in response to various environmental cues. In addition to p53’s established role as a tumor suppressor, it also processes signals from metabolism and autophagy to modulate stemness. For instance, in cancers like hepatocellular carcinoma (HCC), knocking out mitochondrial p53 through mitophagy retains CSCs by preventing p53-mediated inhibition of NANOG and several other stemness genes (Liu et al., 2017). Recent research highlighted that the loss of p53 function upregulates CD44 marker expression, and enhanced stemness in colorectal cancer (Gao et al., 2022). Mutant p53 inhibits autophagic processes by stimulating mTOR or inhibiting AMPK, resulting in increased glycolysis and the Warburg effect, thereby supporting CSC maintenance. AMPK acts as a metabolic sensor that is frequently activated by metabolic stress. In the case of colorectal CSCs there is an increase in AMPK activity levels compared to other cancer cells, which appears to relate to increased mitochondrial biomass and increased ATP synthesis while decreasing ROS (Guo et al., 2018). Inhibition of AMPK with Compound C or Iodotubercidin decreases CSC survival *in vitro*, suggesting that AMPK could be promoting cell survival through mitochondrial biogenesis along with enough NADPH to scavenge ROS (Ratnikov et al., 2015) AMPK activation has also been shown to promote the maintenance of CSCs while AMPK inhibition would eventually sensitize CSC to metabolic stress (Figure 5).

BNIP3 (Bcl-2 interacting protein 3) and its paralog BNIP3L/NIX are hypoxia-induced proteins that have LC3-interacting regions that will bind to the autophagosomal machinery for mitophagy. Breast Cancer cells with high levels of transcription of BNIP3 and BNIP3L from hypoxia have increased lysosome mass, basal mitophagy and sphere-forming capability (Mauro-Lizcano et al., 2024; Liu et al., 2020). These cells also have greater ATP production, increased proliferative and migratory capability, increased antioxidant capacity and drug resistance. These findings suggest BNIP3 increases CSC characteristics via mitophagy in MCF and MDA-MB-231 breast and ovarian cancer cell lines (Mauro-Lizcano et al., 2024; Li et al., 2025). In a similar vein, mitophagy serves energetic needs via removal of dysfunctional mitochondria while avoiding apoptosis and suggests BNIP3 and BNIP3L would be a therapeutic target (Onishi et al., 2021). In addition to the NIX/BNIP3 pathway, there are multiple pathways present in CSCs that utilize mitophagy. The PINK1/Parkin pathway for instance, is activated when mitochondrial membrane potential drops, allowing PINK1 (PTEN-induced kinase 1) to accumulate on the outer mitochondrial membrane and recruit the E3 ligase Parkin and ubiquinate proteins like VDAC1 (Voltage-Dependent Anion Channel 1) (Lin et al., 2021). The ubiquitinated mitochondria are recognized by the adaptor protein p62/SQSTM1, which will link the mitochondria to the LC3 positivity autophagosome and completes breakdown through the lysosome (Tal et al., 2009). Receptor-mediated mitophagy via BNIP3 and or NIX as well as FUNDC1 (FUN14 domain containing 1) occur independently of the ubiquitination process, with these proteins physically binding to LC3 through their LC3-interaction regions, and FUNDC1 is particularly enhanced by hypoxia-induced dephosphorylation to promote interaction with LC3 and mitophagy (Lampert et al., 2019). In summary, a broad array of molecular regulators organizes mitochondrial function in CSCs with respect to biogenesis, dynamics, mitophagy, metabolism, and redox balance. Elucidating these molecular regulators would provide avenues to develop a targeted therapy that will affect mitochondrial dependencies that regulate CSCs while preserving normal stem cells (Table 3).

**TABLE 3:** Represents various molecular players and their mechanisms, impact on CSC function along with therapeutic implications.

Key molecular players	Mechanism of action	Functional impact on CSCs	References
SIRT1, c-Myc, PGC-1α, and p53	1. SIRT1 deacetylates and stabilizes c-Myc, enhancing transcription of mitochondrial biogenesis genes 2. Facilitates nuclear export of p53, relieving suppression of NANOG and maintaining pluripotency	Promotes CSC proliferation, stemness, and resistance to oxidative stress through enhanced mitochondrial metabolism	Tang et al. (2017), Zhao et al. (2023), Li et al. (2014), Kang et al. (2016)
AMPK, mTOR, NADPH, and ROS	1. AMPK senses low energy and activates mitochondrial biogenesis and ROS detoxification pathways 2. High AMPK activity in CSCs correlates with elevated ATP and reduced ROS. 3. AMPK inhibition (Compound C, Iodotubercidin) reduces CSC viability	Maintains metabolic flexibility and survival under stress; supports NADPH production and redox homeostasis	Guo et al. (2018), Ratnikov et al. (2015), Jäger et al. (2007)
BNIP3, BNIP3L, PINK1, Parkin, and p62/SQSTM1	1. BNIP3 and BNIP3L mediate hypoxia-induced mitophagy via LC3-interacting regions 2. PINK1/Parkin pathway ubiquitinates damaged mitochondria for autophagic clearance	Preserves mitochondrial quality, prevents apoptosis, and sustains energy production under hypoxia or nutrient stress	Chinnadurai et al. (2008), Mauro-Lizcano et al. (2024)
NRF2, and FOXO1	1. CSCs maintain low ROS via upregulation of glutathione biosynthesis enzymes and antioxidant transcription factors (NRF2, FOXO1) 2. NRF2 signaling promotes expression of detoxifying enzymes and coordinates with mitophagy to maintain mitochondrial integrity	Ensures redox balance, therapy resistance, and protection against oxidative damage	Dinkova-Kostova and Abramov (2015), Haraguchi et al. (2010), Narayanan et al. (2025), Hayes et al. (2020)

## 6 Mitochondrial dynamics and stemness

In various CSC models, higher fission rates are mostly associated with more aggressive, self-renewing phenotypes, while higher rates of fusion are frequently related to quiescence and differentiation. Recent research shows that cancer cells are often found to have fragmented mitochondria and higher rates of fission are linked with invasiveness, and rapid proliferation. For instance, breast and brain CSCs contain punctate mitochondrial networks, and high DRP1-mediated fission (Xie et al., 2015). Both the fission GTPase DRP1 and its adaptors (MFF and FIS1) are often upregulated or activated in CSCs. In glioblastoma stem-like cells, there are more fragmented mitochondria than differentiated progeny, and DRP1 is hyper-phosphorylated at Ser616. Interestingly, it has been found that DRP1 inhibition leads to apoptosis in these CSCs and a loss of self-renewal capacity (Peiris-Pagès et al., 2018; Kleele et al., 2021). An epigenetic circuit (BRD4 to MFF) regulates overactive mitochondrial fission in metastasis-initiating CSCs in prostate cancer. Inhibition of BRD4 or knockdown of MFF can diminish CSCs self-renewal and tumor progression (Civenni et al., 2019). Likewise, lung and leukemia CSCs rely on fission for protective mitophagy. For example, lung adenocarcinoma CSCs depend on the fission protein FIS1 to trigger mitophagy and maintain stemness. Often elevated FIS1 levels correspond with poorer survival (Liu et al., 2021). Likewise, in AML stem cells, FIS1-mediated mitophagy is necessary for self-renewal. For instance, FIS1-knockdown disrupts mitophagy and promotes differentiation of leukemia stem cells (Pei et al., 2018). The fusion proteins MFN1/2 and OPA1 facilitate formation of interconnected networks which influence numerous processes including metabolism and differentiation. It has also been demonstrated in an EMT model of mammary stem cells that this mechanism stimulates MFN1-mediated fusion that is, MFN1 complexes with polarity proteins, allowing fused mitochondria to be anchored to stem like daughter cells and sustain the renewable stem cell pool (Wu et al., 2019).

In transformed fibroblasts and numerous cancer cells, fusion driven by MFN2 increases OXPHOS for increased proliferation (Yao et al., 2019). In pancreatic ductal adenocarcinoma (PDAC) CSCs, OPA1, associated with inner membrane fusion, is also overexpressed and OPA1 knockdown decreases tumor-sphere formation by half, and reduces predominant stem markers (Carmona-Carmona et al., 2022). Therefore, fusion machinery is often upregulated during the acquisition of stemness in CSCs. For example, PDAC CSCs cultured for prolonged periods have increased gene expression of mitochondrial fusion and biogenesis genes. Additionally, when OPA1 is silenced in these cells, CSC differentiation markers are induced, and these cells lose the ability to form spheres (Courtois et al., 2021). The different behaviors exhibited by CSCs rely on numerous enzymes and pathways. For example, CDK5 activates DRP1 in glioblastoma CSCs, while CaMK2 causes its inactivation (Xie et al., 2015). The tumor suppressor p53-mediated support of fused mitochondria occurs in non-CSCs, but CSCs often exceed this control. Similarly, AMPK signaling is involved with the activation of FIS1 and mitophagy in LSCs to facilitate stemness. Taken together, this key work demonstrates how a few upstream factors (oncogene, cell cycle, metabolic stress) influence the fission/fusion process in determining interesting behaviors of the CSCs. Factioning CSC and non-CSC contacts may be useful for the proper diagnosis and design of treatment strategies.

Additionally, it is important to note that CSC and non-CSC contacts often demonstrate different mitochondrial morphology. The analysis of mitochondrial networks, fission and fusion proteins, can indicate areas of tumor with elevated stem cell densities (Sessions and Kashatus, 2021; Liu et al., 2021). Specifically, the interventions that target these dynamics may have effects on CSCs. Recent research suggests diminished sphere formation and metastasis in various malignancies can be accomplished with DRP1 inhibitors such as mDIVI1 and gene knockdowns (Wang Z. et al., 2024). Fusion targeted therapies may accomplish the reduction of CSC populations when done in combination with ferroptosis-inducing therapies. In contrast, agents that disrupt fusion proteins, such as OPA1 inhibitors, may induce differentiation or death in CSCs. Inhibition of the upstream regulators of dynamics for example, the blockage of the BRD4 to MFF conversion in prostate CSCs, or restoration of the YY2 tumor suppressor to lower DRP1 in liver CSCs also provides additional therapeutic strategies (Civenni et al., 2019; Wei et al., 2023) (Table 4).

**TABLE 4:** A comprehensive table represents key regulators, their mechanisms of action and their impact on stemness.

Regulator	Mechanistic role in CSCs	Impact on stemness
DRP1	Central fission enzyme drives mitochondrial fragmentation and is hyperactivated in CSCs. Its inhibition (For instance by mDIVI1) suppresses tumorsphere formation and mitochondrial-dependent survival	Sustains self-renewal, metabolic flexibility, and tumor-initiating potential
MFF, FIS1 (Fission Adaptors)	Recruit DRP1 to the mitochondrial outer membrane. MFF is transcriptionally upregulated by oncogenic factors (Myc, BRD4). FIS1 promotes mitophagy in lung and AML CSCs to clear damaged mitochondria	Maintain redox balance, enhance metabolic resilience, and preserve CSC maintenance
MFN1, MFN2 (Fusion Proteins)	Mediate mitochondrial outer membrane fusion. MFN1 ensures inheritance of fused mitochondria in stem-like mammary cells, while MFN2 supports OXPHOS and CSC proliferation	Promote metabolic homeostasis and long-term survival under stem-like conditions
OPA1 (Inner Membrane Fusion Protein)	Controls mitochondrial inner membrane fusion and cristae structure. Overexpressed in several CSCs; its depletion disrupts cristae integrity, decreases ATP synthesis, and reduces stemness markers	Preserves mitochondrial architecture, bioenergetic efficiency, and pluripotency programs
CDK5, AMPK, YY2 (Signaling Regulators)	CDK5 phosphorylates DRP1 to promote fission in glioblastoma CSCs. AMPK activates FIS1-mediated mitophagy in AML. YY2 represses DRP1 transcription, counteracting excessive fission in liver CSCs	Regulate mitochondrial morphology and energy balance to fine-tune CSC proliferation and self-renewal

Quiescent or nutrient-deprived CSCs primarily utilize fatty acid oxidation for their growth and maintenance, whereas proliferating CSCs often use glycolysis or pentose phosphate pathway (Kaur and Bhattacharyya, 2021). The difference in metabolism is the basis of the treatment-resistance phenotype: For example, HIF-1α can induce a shift to glycolysis, while MYC or PGC-1α promote OXPHOS and antioxidant activity (Daneshmandi et al., 2019). Multiple therapeutic strategies have been explored, with the goal of disrupting either glycolytic or OXPHOS dependent CSCs, for instance, 2-deoxyglucose and GLUT/hexokinase inhibitors; PDK inhibitors such as dichloroacetate (Michelakis et al., 2008; Liu et al., 2012). For instance, it has been demonstrated that a low dose of metformin blocked mitochondrial complex I, resulting in a low energy situation and leading to apoptosis in CSCs dependent on OXPHOS for energy. Similarly, salinomycin and doxycycline can selectively decrease the viability of CSCs as they prevent ATP synthesis (Gupta et al., 2009). Other such targets include glutaminolysis pathway (glutaminase inhibitors) and lipid synthesis. As such, the coexistence of both glycolytic (proliferating) and mitochondrially dependent (quiescent) CSCs necessitates multi-modal approaches: pairing conventional cytotoxic agents with metabolic inhibitors or combinations of multiple pathway inhibitors may represent a promising approach to curbing the incidence of treatment challenge-induced CSC relapse.

## 7 Therapeutic implications

Targeting mitochondrial function in CSCs represents a transformative strategy to overcome cancer resistance and relapse, based on the unique metabolic and structural dependencies of CSCs on mitochondrial respiration and dynamics (Uslu et al., 2024). Mitochondria serve as central hubs of bioenergetics, biosynthesis, and redox regulation in CSCs, enabling their survival, self-renewal, and evasion of conventional therapies. This therapeutic paradigm leverages vulnerabilities in mitochondrial oxidative phosphorylation, biogenesis, and dynamic remodeling, offering avenues to selectively eradicate CSCs and improve clinical outcomes (Vasan et al., 2020; Snyder et al., 2018).

Dysregulated metabolic reprogramming confers CSCs with the ability to rely predominantly on OXPHOS for ATP generation and to maintain redox homeostasis, especially under the hypoxic and nutrient-poor conditions of the TME. This dependence distinguishes CSCs from bulk tumor cells, which typically favor glycolysis. Pharmacological inhibition of key mitochondrial complexes, primarily Complex I, using agents such as IACS-010759 and phenformin, has demonstrated potent suppression of CSC self-renewal and tumorigenic capacity. These inhibitors exert their effect by impairing electron transport chain function, reducing ATP production, and enhancing oxidative stress, triggering selective apoptosis in CSCs (Zhou et al., 2023). Recent preclinical models highlight that OXPHOS blockade resensitizes resistant cancer variants, including triple-negative breast cancers, to chemotherapeutic regimens and immune checkpoint blockade (Uslu et al., 2025). CSCs across multiple cancer types, including pancreatic ductal adenocarcinoma, lung, glioblastoma, and colon cancer, demonstrate a metabolic dependency on OXPHOS, distinguishing them from the primarily glycolytic bulk tumor cells (Xu et al., 2020). Pharmacologic OXPHOS inhibition induces apoptosis and decreases sphere formation and growth in CSC-enriched cultures, highlighting the therapeutic vulnerability of these cells to mitochondrial inhibitors (Keoh et al., 2025; Narayanan et al., 2025). This approach exploits CSC-specific metabolic vulnerabilities and circumvents limitations of glycolytic inhibition, positioning mitochondrial OXPHOS as a cornerstone therapeutic target.

Mitochondrial biogenesis and dynamics regulate mitochondrial mass, morphology, and quality, which are essential for CSC metabolic plasticity and stress adaptation. The transcriptional coactivator PGC-1α orchestrates mitochondrial biogenesis, while proteins such as DRP1 and MFN2 govern mitochondrial fission and fusion dynamics. Disrupting these pathways through selective inhibitors destabilizes mitochondrial networks, disrupts energy metabolism, and sensitizes CSCs to apoptosis (Abu Shelbayeh et al., 2023). For instance, Mdivi-1, a DRP1 inhibitor, inhibits mitochondrial fission, augmenting mitochondrial dysfunction in CSCs (Rosdah et al., 2022). Suppressing PGC-1α signaling impairs biogenesis, reducing mitochondrial abundance and metabolic flexibility. Novel molecules (DRP1i27) and repurposed drugs (doxycycline, mTOR inhibitors) expand the arsenal to target CSC mitochondrial integrity effectively, thereby limiting tumor progression and relapse (Table 5).

**TABLE 5:** Inhibitors of mitochondrial biogenesis and dynamics in cancer stem cells.

Inhibitor/Strategy	Molecular target	Mechanism of action	Effect on CSCs	References
Mdivi-1	DRP1	Inhibits mitochondrial fission, destabilizing networks	Induces CSC apoptosis, impairs renewal	Silva-Pavez et al. (2024)
DRP1i27/Drpitors	DRP1	Potent GTPase domain inhibitors; block fission	Reduce proliferation; induce apoptosis	Rosdah et al. (2022)
SR18292/siRNA	PGC-1α	Suppress biogenesis, affecting mitochondrial mass and function	Lower CSC metabolic plasticity; reduce tumorigenic potential	Sharabi et al. (2017)
XCT790	ERRα-PGC-1α pathway	Blocks biogenesis signaling, reduces OXPHOS activity	Suppresses CSC survival and propagation	De Luca et al. (2015)
Doxycycline	Biogenesis-related proteins	Repurposed antibiotic, inhibits mitochondrial biogenesis proteins	Prevents tumor recurrence via FOXM1 and mitochondrial suppression	Scatena et al. (2018)
mTOR inhibitors (Rapamycin)	mTOR	Decrease biogenesis via mTOR pathway inhibition	Sensitize CSCs to therapy	Panwar et al. (2023)

Combining mitochondrial-targeting agents with chemotherapy or immunotherapy has shown remarkable promise in enhancing antitumor efficacy by targeting resilient CSCs and overcoming therapeutic resistance (Table 6). Co-administration of mitochondrial OXPHOS inhibitors with chemotherapeutics like cisplatin and paclitaxel depletes CSC reservoirs and disables tumor-initiating capacities (Keoh et al., 2025). Similarly, integration with immune checkpoint inhibitors (anti-PD-1/PD-L1) potentiates immunogenic cell death, activates cytotoxic immune responses, and reprograms the TME from immunosuppressive to immunostimulatory. Advanced modalities such as mitochondrial-targeted photothermal therapies further augment these effects by inducing localized mitochondrial damage (Zheng et al., 2025). This multi-pronged approach effectively abrogates CSC-mediated immune evasion and facilitates long-lasting cancer remission.

**TABLE 6:** Combinatorial therapies incorporating mitochondrial-targeting agents with chemotherapy and immunotherapy in cancer stem cells.

Combination	Agents/Inhibitors	Cancer models	Effects/Mechanisms	References
OXPHOS inhibitors + Cisplatin/Paclitaxel	IACS-010759, phenformin + cisplatin/paclitaxel	Various solid tumors, CSC-rich	Depletes CSCs, enhances apoptosis, impairs EMT	Skoda et al. (2019), Pujalte-Martin et al. (2024)
Nanoparticle-PTT + Checkpoint Inhibitor	Nano-PTT/CDT + anti-PD-1	Colon cancer (mouse)	Mitochondrial damage, dendritic/CD8+ T cell activation	Zheng et al., 2025
Mito-FF + Paclitaxel	Mitochondrial agent + paclitaxel	Breast cancer	ETC/microtubule disruption, increased apoptosis	Ahn et al., 2025
Mito-CP/Mito-Q + 2-DG	Mitochondrial electron transport inhibitors + glycolytic	Breast cancer	Dual pathway cytotoxicity against CSCs	Cheng et al., 2012
Pro-oxidant Mito. Targeting + Chemo	Orthomolecular drugs + standard chemotherapy	CSC models	ROS-mediated selective CSC apoptosis, protection for healthy SC	Mani et al., 2022
Mitochondrial Targeting + Immunotherapy	Organic mitochondrial sensitizers + immune checkpoint	Immunotherapy models	Increased immune activation via ROS modulation	Jing et al. (2025)

Despite promising therapeutic advances, selective targeting of mitochondrial function in CSCs remains challenging. Key hurdles include minimizing off-target toxicity to normal stem and progenitor cells, overcoming CSC metabolic plasticity that enables switching between OXPHOS and glycolysis, and addressing tumor heterogeneity with metabolically diverse CSC subpopulations (Garimella et al., 2023). Additionally, efficient delivery of mitochondrial inhibitors to CSC niches is impeded by drug bioavailability and penetration barriers. Resistance mechanisms and metabolic adaptation necessitate rational combination or sequential therapeutic regimens guided by biomarker-driven patient stratification (Lee et al., 2025). Innovations in targeted nanodrug delivery and metabolic profiling are critical to improving specificity and overcoming current therapeutic limitations (Pandya et al., 2023).

## 8 Diagnostic and prognostic potential

Mitochondrial and metabolic reprogramming form a core biology of CSCs, supporting initiation, therapeutic resistance, and recurrence of tumors. Among various functional readouts, ALDH activity remains one of the robust hallmarks for the enrichment of CSCs across HNSCC and pancreatic ductal PDAC. High expression of ALDH1A1 or ALDEFLUOR™ activity portends poor patient survival and is associated with resistance to radiation and chemotherapy (Ginestier et al., 2007). Enzymes governing FAO and oxidative OXPHOS, such as CPT1A, PGC-1α, and TOMM20, reflect mitochondrial mass and respiratory capacity and SIRT3/5 maintain redox homeostasis (Nimmakayaoa et al., 2021). These nodes together define a metabolic axis that sustains CSCs persistence (Du et al., 2025; Sancho et al., 2015; Viale et al., 2014; Islam et al., 2025; Melcher et al., 2017).

Transcriptomic OXPHOS signatures incorporating ETC subunits including NDUFS, UQCRC, COX which have been aggregated into OXPHOS high scores that stratify patient outcomes and correlate with metastasis. Simultaneously, redox-detox enzymes such as PRDX3/5 and TXN2, along with ALDH1A1, link mitochondrial defense to the maintenance of stemness. At the genomic-organelle level, mtDNA copy number, heteroplasmy patterns, and mitochondrial-membrane potential (ΔΨm) are considered measurable CSC indicators. Hyperpolarized ΔΨm and increased mtDNA burden have been observed in aggressive subclones.

Complementing these findings, protein, and surface markers point out that CD44, especially CD44v9, in combination with CD133 and EpCAM, when co-stained with PGC-1α or TOMM20, delineate metabolic CSCs (Hirata et al., 2021; Pastò et al., 2014). Nutrient-transporter expressions, including MCT1 (Mono carboxylase transporter 1) and ASCT2 (Alanine-serine-cysteine Transporter 2), can be used to further report tumor metabolic dependencies.

Emerging imaging approaches now enable *in vivo* quantification of CSC metabolism. Hyperpolarized ^13^C-MRI spectroscopy using [1-^13^C] pyruvate yields real-time maps of lactate/pyruvate flux, distinguishing glycolytic versus OXPHOS phenotypes and yielding an early marker of response to metabolic inhibitors (Kurhanewicz et al., 2019). Clinically established PET tracers-^18^F-FDG (glycolysis), ^11^C-acetate (lipid synthesis/OXPHOS tilt), and ^18^F-fluoroglutamine (glutamine anaplerosis) extend this capacity to diverse solid tumors (Momcilovic et al., 2019). In research settings, two-photon FLIM of NADH/FAD ratios or MRI-based CEST contrasts yield complementary pH and redox information, revealing metabolic heterogeneity within tumor sections.

Spatial metabolomics, particularly MALDI-MS imaging, maps metabolic gradients like OXPHOS-high pockets rich in acyl-carnitines and TCA intermediates and tumor infiltration *in situ*, notably demonstrated in brain cancer surgical specimens (Ji et al., 2015). Their integration with multiplex IHC/IF for CD44/ALDH1 and mitochondrial markers helps in identifying sub-regions related to radio-resistance (Shakyawar et al., 2023). Combining metabolic imaging, hypoxia maps, and CAF density within mixed-effects models improves the performance for predicting progression-free survival and overall survival (Zhi et al., 2024).

Liquid-biopsy approaches further democratize access to CSC biomarkers. Circulating cell-free mtDNA levels and integrity indices correlate with tumor burden and therapeutic response, while exosomal cargoes, such as ALDH1A1, PGC-1α, PKM2, and MCT1, reflect mitochondrial stress signaling (Haldavnekar et al., 2022; Gening et al., 2021; Heidrich et al., 2021; Lim et al., 2019; Wang et al., 2023). Metabolites like acylcarnitines, glutamine/glutamate, and lactate/pyruvate ratios complement genomic data to yield dynamic disease readouts. CTC phenotyping by flow cytometry or immunofluorescence identifies ALDHhigh/ΔΨmhigh circulating CSCs tracking metastatic potential (Tang et al., 2024). These omics-based assays can evolve into multiplex clinical panels that combine cf-mtDNA integrity, exosomal PGC-1α, and plasma metabolite signatures for real-time treatment monitoring.

## 9 Conclusions and future perspectives

In recent years, research has revealed that mitochondria plays a crucial role in regulating CSC biology, forming the link between bioenergetics and the stem cell-like phenotype. CSCs often reprogram metabolic pathways to fulfill the cellular demands for optimal proliferation and redox. While CSCs generally depend on glycolysis, they also engage in the dynamic mitochondrial OXPHOS machinery to meet energetic demand (Garimella et al., 2023). Elevated mitochondrial respiration promotes resistance to depletion and oxidation of CSCs while maintaining elevated stress, self-renewal, and drug resistance. Mitochondrial ROS and metabolites can also serve as signaling molecules that influence nuclear gene expression and are subject to epigenetic regulation, further providing characteristics necessary for CSC identity and function. Metabolic rewiring that supports a bioenergetic demand, in response to depletion in nutrient or oxygen conditions present in the TME, is relevant especially to metastasis and the resistance to targeted therapies (Liu and Wang, 2024).

Molecular factors that regulate mitochondrial dynamics are also critical for either establishing or maintaining CSC identity. For instance, Mitochondrial biogenesis driven by PGC-1α was determined to be necessary to support OXPHOS activity, and associated tumorigenic potential, in CD133^+^ pancreatic CSCs (Sancho et al., 2016). Notably, mitochondrial-nuclear retrograde signaling induced by ROS or metabolite-based signals can trigger activation of transcription that promote survival and EMT, thus supporting the persistence of CSC properties. These notions support a rationale for mitochondria-directed therapy: metabolic agents such as ETC inhibitors can exert selective collapse of CSC mitochondrial function that can lead to apoptosis.

The next Frontier to explore is single-cell resolution of metabolic heterogeneity. Integrative multiomics in combination with scRNA seq, acATCC seq, and scProteomics with optical readouts including FLIM of NADH/FAD ratios or stable isotope tracing on micro samples will allow the discrimination between OXPHOS high and Glycolytic CSC substrates (Zhang et al., 2024). Spatial multi-omics, i.e., MALDI/DESI MSI coupled with CODEX or IMC immunophenotyping, will chart the topography of CSC niches along the oxygen, pH, and stromal gradients *in situ*. Longitudinal sampling of patient biopsies before and during therapy will capture dynamic metabolic switching events prior to clinical relapse.

Metabolic profiling will showcase patient stratification and rational combinations. Tumors harboring OXPHOS-high/FAO-dependent CSCs may benefit from Complex I inhibitors-IACS-010759, FAO modulators such as CPT1 blockers, or DHODH inhibitors such as brequinar or leflunomide (Molina et al., 2018; Bajpai et al., 2020). Glutamine-addicted CSCs respond to glutaminase inhibitors like telaglenastat (CB-839) combined with mTOR blockade. Pairing mitochondrial inhibitors with DNA-damage response agents-PARP or ATR inhibitors-could exploit ROS/DNA repair liabilities, while coupling to immune checkpoint blockade may re-educate the tumor microenvironment metabolism. Biomarker-driven response metrics include a decrease in the HP-^13^C lactate/pyruvate ratio, a reduction in ΔΨm in CTCs, normalization of cf-mtDNA, and a collapse of OXPHOS signatures that offer quantitative endpoints for adaptive trials.

Further, a three tier pipeline will facilitate the bench to bedside transition including discovery and analytical validation of candidate markers, including ALDH activity, OXPHOs genes and cf-mt DNA integrity. Further the targeted panels could be developed using MRM/PRM mass spectroscopy or ddPCR for cf-mtDNA. These techniques further could be integrated with the liquid biopsy monitoring and spatial metabolic state of each tumor. Mitochondrial and metabolic biomarkers are well positioned to change the face of CSC-focused diagnostics and therapeutics in solid tumors.

In summary, both metabolic and mitochondrial biomarkers offer a quantitative and clinically traceable window into CSC state, plasticity and therapeutic vulnerability. Integrating these signatures with real time imaging, liquid biopsies, and next-generation organoid models will not only remain risk stratification but will also enable mechanism, guided, patient specific treatment selection. The convergence of CSC metabolism, precision diagnosis, and targeted therapy thus represents a rapidly maturing Frontier that can redefine outcomes in solid tumors.
